# Impact of Modifiable Risk Factors on the Occurrence of Cutaneous Leishmaniasis in Diyala, Iraq: Case-Control Study

**DOI:** 10.2196/28255

**Published:** 2021-07-30

**Authors:** Asaad Mahdi Lehlewa, Hanan Abdulghafoor Khaleel, Faris Lami, Saif Aldeen Falah Hasan, Hasanain Asmail Malick, Razzaq Hashim Mohammed, Qais Abdulazziz Abdulmottaleb

**Affiliations:** 1 Public Health Directorate Ministry of Health of Iraq Baghdad Iraq; 2 Communicable Diseases Control Center Public Health Directorate Ministry of Health of Iraq Baghdad Iraq; 3 Department of Community and Family Medicine College of Medicine University of Baghdad Baghdad Iraq; 4 Ministry of Health of Iraq Baghdad Iraq; 5 Ministry of Health of Iraq Babylon Iraq

**Keywords:** cutaneous leishmaniasis, outbreak, Iraq, risk factors, risk, disease, infectious disease, disease prevention, prevention

## Abstract

**Background:**

In 2018, an outbreak of cutaneous leishmaniasis (CL) occurred in Diyala Province in Iraq. Several risk factors of CL were identified in a prior study; however, the impact of removing modifiable risk factors on the occurrence of the disease was not measured.

**Objective:**

The aim of this study is to measure the impact of removing modifiable risk factors of CL on the occurrence of the disease.

**Methods:**

We conducted a population-based unmatched case-control study in two conveniently selected districts in Diyala Province. All cases of CL were included. Controls were chosen preferentially according to the site where the cases occurred. A structured questionnaire was used to collect data. The unadjusted odds ratios (ORs) and 95% confidence intervals for each risk factor were calculated using binary logistic regression. We also calculated the attributable fractions and 95% confidence intervals of the modifiable risk factors. A *P* value <.05 was considered statistically significant.

**Results:**

Data from 844 persons (432 cases, 51.2%) were analyzed. Cases were more likely than controls to report a history of previous displacement (OR 5.18, 95% CI 3.84-6.98), electricity supply for less than 12 hours per day (OR 1.94, 95% CI 1.47-2.55), living in a rural area (OR 1.91, 95% CI 1.45-2.51), living in a clay house (OR 2.41, 95% CI 1.59-3.66), having an unpainted indoor living space (OR 2.14, 95% CI 1.51-3.02), having rodents inside the house (OR 5.15, 95% CI 3.56-7.47), having chickens, sheep, or both (OR 3.44, 95% CI 2.48-4.75), having a mixture of dogs and sheep or of dogs and chickens within a distance of less than 100 meters (OR 3.92, 95% CI 2.59-5.94), fogging (OR 2.11, 95% CI 1.40-3.19), bed net use (OR 1.72, 95% CI 1.08-2.72), and sleeping outside or a mixture of inside and outside (OR 4.01, 95% CI 1.32-12.19). The data show that the exposure of approximately 70% to 80% of cases was associated with displacement, the presence of rodents inside the house, the presence of animals within 100 meters of the house, the presence of animals (chickens/sheep/both or a mixture of dogs and sheep or of dogs and chickens), and sleeping outside. Approximately 40%-50% of the cases reported living in a clay house, living in a rural area, having an unpainted indoor space, having an electricity supply for less than 12 hours, and using a bed net.

**Conclusions:**

Prevention and control of CL requires a multifaceted approach that relies on changing environmental conditions, housing conditions, and human behavior. Fogging and bed net use were not effective because the underlying housing characteristics and human behavior provided a good culture for the disease. We recommend conducting a study to identify the species, reservoirs, and vectors of CL in Iraq; studying vector behaviors before applying environmental control measures; and educating the public on how and when to use bed nets as well as how to accompany their use with behavioral changes.

## Introduction

Cutaneous leishmaniasis (CL) is a neglected tropical disease for which approximately 500,000 to 1,000,000 new cases are reported per year worldwide [1,2]. Furthermore, it causes an estimated 2.4 million disability-adjusted life years, placing it among the top 10 in a global analysis of infectious diseases [3]. Countries in the Eastern Mediterranean region contribute approximately 57% of the total CL burden, where *Leishmania tropica* and *Leishmania major* are endemic in 18 countries and territories (including Iraq). Moreover, more than 100,000 new cases of CL are reported annually to the World Health Organization by countries in the Eastern Mediterranean region; however, the actual incidence is estimated to be 3 to 5 times higher [1,4,5]. In Iraq, surveillance data after the 1970s showed an average of 10x00 cases per year [6]. According to internal technical reports released by the Iraqi Ministry of Health, the last country-wide outbreak started at the end of 2014 and continued throughout 2017, when the number of cases per year reached an average of 16,000. In 2018, the number of cases started to decline steadily and reached approximately 11,000.

There are more than 20 *Leishmania* species that can be transmitted to humans, and more than 90 sand fly species that can transmit the protozoa to humans; moreover, approximately 70 animal species, including humans, are natural reservoir hosts of *Leishmania* parasites [7]. The transmission cycle of the parasite in nature can be either zoonotic or anthroponotic [8,9]. In Iraq, data are lacking regarding the most common *Leishmania* species, reservoirs, and vectors. However, evidence from nearby countries suggests that both transmission cycles of CL (zoonotic and anthroponotic) are common in Iraq [5,10,11].

Risk factors for developing CL include residence in rural areas, climate changes, movement of people, conflict areas, deforestation, house characteristics, and human behavior [9,12-14]. Prevention and control of leishmaniasis requires a combination of intervention strategies because transmission occurs in a complex biological system involving the human host, parasite, sand fly vector, and, in some cases, an animal reservoir host. Key strategies for prevention are early diagnosis and effective case management, vector control, effective disease surveillance, control of animal reservoir hosts, and social mobilization and strengthening partnerships among all concerned institutions [14].

Although CL is a self-healing disease, it is potentially disfiguring [1]. The only drug licensed by the Iraqi Ministry of Health to treat CL is sodium stibogluconate, a pentavalent antimony compound.

The recent outbreak affected most Iraqi provinces variably, with an overall incidence rate of 0.9/10^3^ population. The highest incidence rate was in Diyala Province (4/10^3^ population), while the lowest incidence rate was in Duhok Province (0.01/10^3^ population). According to internal reports and discussion with the zoonotic diseases section at the Iraq Communicable Diseases Control Center, the lack of infrastructure and municipal services, the presence of hard-to-reach areas, and a lack of prevention programs were blamed for the occurrence of the outbreak. Diyala was subjected to terrorist and military operations from 2014 to 2016, when most of its residents were displaced. Meanwhile, it also encountered a wave of a *Leishmania* epidemic that started in November 2014, reached its peak during 2015, and continued throughout 2017. In response to the rapid escalation of the outbreak, the outbreak response team investigated the outbreak to identify possible risk factors and the impact of removing these factors on reducing the number of cases.

## Methods

This is a population-based unmatched case-control study. A case of CL was defined as any person who showed clinical signs (skin or mucosal lesions) and was diagnosed by a dermatologist with CL. A control person was defined as any person (or family member) who was proved to be free of these skin or mucosal lesions. Controls were chosen preferentially according to the site where the cases occurred (from the neighboring house or village). The study was conducted in two conveniently selected districts in Diyala Province (Al-Muqdadiya and Al-Mansuriya). Those two districts were selected as part of the on-job outbreak investigation because surveillance data detected an increase in the number of CL cases in these areas, and those areas were in the recovery process after security instability. Approval for conducting the study was obtained from the Public Health Directorate/Ministry of Health and Diyala Directorate of Health. Oral consent was obtained from the cases and controls themselves or from their caretakers.

Field epidemiology training program students interviewed cases and controls using a modified questionnaire of the case investigation form of the zoonotic section of the Iraq Communicable Diseases Control Center. The questionnaire contained questions about the main demographic (age, sex, occupation), clinical (date of onset, signs and symptoms, presence of other cases within the family, treatment, previous visits, number and site of skin lesions), and epidemiological characteristics (displacement history, house and residency data [information about the type of residency area; house construction materials, such as wall type; electricity provided; animals living within the house; painting of indoor areas; presence of rodents inside or around the house]), sleeping habits, and preventive measures implemented in the area (fogging and use of bed nets).

A total of 866 persons were interviewed within the 717 families visited: 451 cases (292 from Al-Mansuriya District and 159 from Al-Muqdadiya District) and 415 controls (182 Al-Mansuriya District and 233 from Al-Muqdadiya District). However, we excluded 22 persons from the sample due to incomplete information. The final sample size used was 844 persons (cases=432, controls=412), with a ratio of almost 1 case to 1 control.

Univariate analysis was used to describe the study sample. Bivariate analysis was used to detect possible associations between each of the risk factors and the disease (CL) using the chi-square test of independence. The unadjusted odds ratio (OR) and 95% confidence interval of each risk factor were calculated using binary logistic regression. The attributable fractions and their corresponding 95% CIs were calculated for the modifiable risk factors. A *P* value <.05 was considered statistically significant.

Epi Info, version 7.2 was used for data entry and SPSS, version 25 (IBM Corporation) was used for data analysis.

## Results

Data from 844 persons (432 cases, 51.2%) were analyzed. There were no gender differences between cases and controls. Cases were more likely than controls to report a history of previous displacement, electricity supply for less than 12 hours per day, and living in a rural area. Regarding house characteristics, cases were more likely than controls to report living in a clay house, living in unpainted indoor areas, and the presence of rodents inside the house. As for animal ownership and the distances of the animals from the house, cases were more likely than controls to have chickens only, sheep only, or both and a mixture of animals (dogs and sheep or dogs and chickens) within a distance of less than 100 meters. Regarding possible preventive measures, cases were more likely to report fogging, bed net use, and sleeping outside or a mixture of inside and outside than controls.

Almost all the risk factors were statistically significantly associated with higher odds of having CL. Nevertheless, the strength of the association varied, as it was stronger (4 to 5 times higher odds of having CL) for factors such as displacement, having animals within 100 meters of the house, and sleeping outside the house. Factors that were associated with a 2 to 3 times increase in the odds of having CL included living in a clay house, having an unpainted indoor area, sleeping in a mixed pattern (inside and outside the house), having animals (whether chickens only, sheep only, or both, or mixtures of dogs and sheep or dogs and chickens), and, interestingly, using a bed net and fogging/unknown fogging status. In fact, the use of a bed net was associated with 72% higher odds of having CL in comparison to the lack of use of a bed net (OR 1.72, 95% CI 1.08-2.72). Likewise, fogging and unknown fogging status were associated with statistically significant 2-fold higher odds of having CL compared to no fogging (*P*<.001).

Regarding the impact of removing modifiable risk factors, our results show that approximately 70% to 80% of the cases were associated with displacement, the presence of rodents inside the house, the presence of animals within 100 meters of the house, the presence of animals (whether chicken only/sheep only/both or a mixture of dogs and sheep or dogs and chickens), and sleeping outside. Similarly, approximately 40% to 50% of the exposure of the cases was associated with living in a clay house; living in a rural area; having an unpainted indoor space; having an electricity supply for less than 12 hours per day; and, interestingly, using a bed net. Unexpectedly, approximately 10% to 20% of the exposed cases reported fogging or unknown fogging status. That is, fogging and unknown fogging status were negatively associated with the occurrence of CL.

The characteristics of the study sample are shown in Table 1. The risk factors for CL in the study population are summarized in Table 2.

**Table 1:** Characteristics of the study sample.

Characteristic			Total (N=844), n (%)	Cases, (n=432, 51.2%), n (%)	Controls (n=412, 48.8%), n (%)	*P* value
**Demographics**						
	**Age group (years)**					<.001
		<15	607 (71.9)	358 (82.9)	249 (60.4)	
		≥15	237 (28.1)	74 (17.1)	163 (39.6)	
	**Gender**					.74
		Male	437 (51.8)	226 (52.3)	211 (51.2)	
		Female	407 (48.2)	206 (47.7)	201 (48.8)	
	**Residency**					<.001
		Rural/semiurban	464 (55)	271 (62.7)	193 (46.8)	
		Urban	380 (45)	161 (37.3)	219 (53.2)	
**Characteristics**						
	**Previous displacement**					<.001
		Yes	493 (58.4)	332 (76.9)	161 (39.1)	
		No	351 (41.6)	100 (23.1)	251 (60.9)	
	**Building material of the house**					<.001
		Clay	117 (13.9)	81 (18.8)	36 (8.7)	
		Block/brick	727 (86.1)	351 (81.3)	376 (91.3)	
	**Indoor space**					<.001
		Not painted	178 (21.1)	117 (27.1)	61 (14.8)	
		Painted	666 (78.9)	315 (72.9)	351 (85.2)	
	**Electricity supply (hours per day)**					<.001
		<12	394 (46.7)	236 (54.6)	158 (38.3)	
		≥12	450 (53.3)	196 (45.4)	254 (61.7)	
	**Animals**					<.001
		Dogs only	14 (1.7)	8 (1.9)	6 (1.5)	
		Chickens only/sheep only/both	254 (30.1)	169 (39.1)	85 (20.6)	
		Mixture of dogs and sheep or dogs and chickens	134 (15.9)	93 (21.5)	41 (10)	
		No animals	442 (52.4)	162 (37.5)	280 (68)	
	**Distance of animals from house (meters)^a^**					<.001
		All	436 (51.7)	301 (69.7)	135 (32.8)	
		<100	305 (70)	253 (84.1)	52 (38.5)	
		100-300	111 (25.4)	39 (13)	72 (53.3)	
		>300	20 (4.9)	9 (3)	11 (8.1)	
	**Presence of rodents in the house**					<.001
		Yes	648 (76.8)	388 (89.8)	260 (63.1)	
		No	196 (23.2)	44 (10.2)	152 (36.9)	
	**Use of fogging**					<.001
		Yes	127 (15)	85 (29)	42 (10.2)	
		Unknown	220 (26.1)	104 (24.1)	116 (28.2)	
		No	497 (58.9)	243 (56.3)	254 (61.7)	
	**Use of bed net**					<.001
		Yes	108 (12.7)	55 (12.7)	53 (12.8)	
		Unknown	127 (15)	67 (15.5)	60 (14.5)	
		No	609 (72.3)	310 (71.7)	299 (72.5)	
	**Sleeping habits**					<.001
		Inside the house	668 (79.1)	318 (73.6)	350 (84.9)	
		Outside the house	19 (2.2)	15 (3.6)	4 (1)	
		Inside/outside the house	157 (18.6)	99 (22.9)	58 (14.1)	

^a^Percentages in this category are calculated based on the “All” values.

**Table 2:** The odds ratios, attributable fractions, and 95% confidence intervals of the modifiable risk factors.

Risk factor		Odds ratio (95% CI)	Attributable fraction (%) (95% CI)
Displacement		5.18 (3.84 to 6.98)	80.6 (73.7 to 85.8)
Clay house		2.41 (1.59 to 3.66)	58.5 (36.7 to 72.7)
Residence in rural region		1.91 (1.45 to 2.51)	47.6 (31 to 60.1)
Unpainted interior		2.14 (1.51 to 3.02)	53.3 (33.8 to 66.9)
Electricity for <12 hours per day		1.94 (1.47 to 2.55)	48.30 (31.9 to 60.8)
**Animals**			
	Dogs only	2.30 (0.79 to 6.76)	56.5 (–28.2 to 85.2)
	Chickens only/sheep only/both	3.44 (2.48 to 4.75)	70.9 (59.7 to 78.9)
	Mixture of dogs and sheep or dogs and chickens	3.92 (2.59 to 5.94)	74.5 (61.4 to 83.2)
**Distance of animals from the house (meters)**			
	<100	5.95 (2.35 to 15.07)	83.2 (57.4 to 93.4)
	100-300	0.66 (0.25 to 1.73)	–51.5 (–3 to 42.1)
Presence of rodents in the house		5.15 (3.56 to 7.47)	80.6 (71.9 to 86.6)
**Use of fogging**			
	Yes	2.11 (1.40 to 3.19)	52.6 (28.6 to 68.6)
	Unknown	2.25 (1.43 to 3.56)	55.5 (30.1 to 71.9)
**Use of bed net**			
	Yes	1.72 (1.08 to 2.72)	41.9 (7.4 to 63.2)
	Unknown	1.49 (0.86 to 2.60)	32.9 (–16.3 to 61.5)
**Sleeping habits**			
	Outside the house	4.01 (1.32 to 12.19)	75.1 (24.2 to 91.8)
	Inside/outside the house	2.07 (1.43 to 3)	51.7 (30.1 to 66.7)

## Discussion

### Principal Findings

To our knowledge, this is the first large population-based case-control study performed in Iraq to determine the risk factors of CL and the impact of changing modifiable risk factors. We identified the main domestic and behavioral characteristics associated with increasing the odds of contracting CL, which provides a guide for preventive and control measures.

The main modifiable risk factors were displacement, having animals within 100 meters of the house, and sleeping outside the house. In fact, the exposure of 70% to 80% of the cases was associated with displacement, animals in the house, animals within 100 meters of the house, and sleeping outside. In contrast, preventive measures, such as bed net use and fogging, were not successful in preventing CL, as both were associated with increased odds of having CL. In fact, assuming a causal relationship and no bias, the data show that approximately 42% of the cases who used a bed net and 10% of the cases who reported fogging would not have contracted CL if they had not used bed nets or fogging. This finding could be explained by inappropriate timing of fogging, that is, fogging occurred after people returned to liberated areas and had already been bitten by sand flies. In addition, fogging may have been performed in the afternoon, when sand flies are inactive, and the flies were consequently not affected. Bed net use was also not an effective measure of preventing CL, possibly because the patients went to bed late, when the sand flies were not active, and therefore had already been bitten.

The findings in our study regarding displacement, poor housing conditions, and sleeping outside the house agree with findings from studies of risk factors in developing and developed countries [15],[16] (retracted), [17]. Displacement increases individuals’ risk of exposure to environmental and personal risk factors of developing CL. In addition, areas from which people are displaced, usually war zones, provide a suitable culture for the growth of both vectors and reservoirs of CL because of the accumulation of wastes and the destruction of infrastructure, such as sewage systems [7]. These findings suggest that preventing CL requires a multifaceted approach that focuses on modifying environmental, domestic, and peridomestic characteristics and on changing human behaviors. Our findings are similar to findings from studies of risk factors of CL in Morocco [18,19], Spain [20], and Ghana [20].

Our study has several strengths. First, it is the first large population-based case-control study of a leishmaniasis outbreak. We identified the main risk factors and their attributable fractions, providing an estimate of the public health impact of the disease. In addition, the findings from our study help to guide preventive and control measures as to the timing of fogging, keeping animals outside houses, painting indoors, and sleeping inside houses.

Our study also has a few limitations. First, the duration of the study was limited, as all data were collected in only 4 days; this led to missing information for some of the variables in the original sample, and they were thus excluded. Second, two important variables were missed, namely, time of fogging and time of sleep, which led us to hypothesize that both actions were undertaken at the wrong time and consequently both surfaced as risk factors rather than preventive factors for the disease. Third, the hazardous security situation limited the movement of the team to only safe areas, which could have obscured other risk factors we are not aware of. Finally, no species were identified from the patients, reservoirs, or vectors to establish the linking of the transmission cycle; therefore, the link is only epidemiologic. None of these limitations could have affected findings from our study; nevertheless, they are worth mentioning to direct future studies in Iraq regarding variables to consider.

### Conclusions and Recommendations

CL is an important public health problem in Iraq, especially in Diyala Province. Most of the cases in our study could have been prevented if they were not exposed to displacement, animals inside the house, animals within 100 meters of the house, or rodents in the house. In addition, the timing of fogging and using bed nets is an important consideration. Prevention and control of CL require a multifaceted approach that relies on changing environmental conditions, housing conditions, and human behavior. Fogging and bed net use were not effective because the underlying housing characteristics and human behavior provided a good culture for the disease.

We recommend conducting a study to identify the species, reservoirs, and vectors of CL in Iraq, studying vector behaviors before applying environmental control measures, and educating the public on how and when to use bed nets and accompany their use with behavioral changes, such as using insect repellents and wearing long sleeves. Furthermore, we recommend studying vector and reservoir behaviors before implementing control measures. In addition, we recommend implementing preventive measures, such as fogging and rodent control, in abandoned areas before people resettle after displacement.

## Abbreviations

CL: cutaneous leishmaniasis
EMPHNET: Eastern Mediterranean Public Health Network
OR: odds ratio
